# Supplementary material for: Fascin structural plasticity mediates flexible actin bundle construction
Source: Nat Struct Mol Biol. 2025 Jan 20;32(5):940–52. doi: 10.1038/s41594-024-01477-2 (PMC12086090; doi:10.1038/s41594-024-01477-2)
Supplement: Supplementary file 1 — Reporting Summary [file 41594_2024_1477_MOESM1_ESM.pdf]

nature portfolio

Corresponding author(s): Gregory Alushin
Rui Gong

Last updated by author(s): Sep 12, 2024

Reporting Summary

Nature Portfolio wishes to improve the reproducibility of the work that we publish. This form provides structure for consistency and transparency in reporting. For further information on Nature Portfolio policies, see our Editorial Policies and the Editorial Policy Checklist.

Statistics

For all statistical analyses, confirm that the following items are present in the figure legend, table legend, main text, or Methods section.

n/a	Confirmed	
☐	☒	The exact sample size (*n*) for each experimental group/condition, given as a discrete number and unit of measurement
☒	☐	A statement on whether measurements were taken from distinct samples or whether the same sample was measured repeatedly
☐	☒	The statistical test(s) used AND whether they are one- or two-sided<br>*Only common tests should be described solely by name; describe more complex techniques in the Methods section.*
☒	☐	A description of all covariates tested
☐	☒	A description of any assumptions or corrections, such as tests of normality and adjustment for multiple comparisons
☐	☒	A full description of the statistical parameters including central tendency (e.g. means) or other basic estimates (e.g. regression coefficient) AND variation (e.g. standard deviation) or associated estimates of uncertainty (e.g. confidence intervals)
☐	☒	For null hypothesis testing, the test statistic (e.g. *F*, *t*, *r*) with confidence intervals, effect sizes, degrees of freedom and *P* value noted<br>*Give P values as exact values whenever suitable.*
☒	☐	For Bayesian analysis, information on the choice of priors and Markov chain Monte Carlo settings
☒	☐	For hierarchical and complex designs, identification of the appropriate level for tests and full reporting of outcomes
☒	☐	Estimates of effect sizes (e.g. Cohen's *d*, Pearson's *r*), indicating how they were calculated

Our web collection on statistics for biologists contains articles on many of the points above.

Software and code

Policy information about availability of computer code

Data collection | Cryo-EM and Cryo-ET data were collected with SerialEM v3.8.

Data analysis

For cryo-EM analysis, direct detector frame series were aligned with MotionCor2 v1.3.1, and CTF estimation was performed with CTFFIND4 v4.1.14. With the exception of 2D classifcation, ab initio reconstruction, and initial refinement performed with cryoSPARC v4.2.1, all other steps were performed with RELION4.0.

For cryo-ET analysis, frame alignment and CTF estimation was performed with WARP v1.0.9. Tilt series alignment and reconstruction was performed with IMOD v4.11. Subtomogram averaging analysis was performed with RELION4.0.

Custom code was used for particle picking and denoising / semantic segmentation of cryo-ET data, making use of libraries from EMAN2 v2.91 implemented in Python v3.7.9. This code was generated with the assistance of ChatGPT4.0. Custom code was also used for simulations, implemented in Matlab2022b. All custom code is available as open source at https://www.github.com/alushinlab/fascin

Coot v0.9.2, ISOLDE v1.3, and Phenix v1.19.2 were used for atomic model building and refinement, while Molprobity v4.4 was used for validation. Chimera and ChimeraX v1.6.1 were used for molecular graphics, as well as structural analysis. DynDom was used to analyze domain motions.
The consensus rise and twist of actin filament was measured using both the relion_helix_toolbox in RELION 4.0 and the Iterative Helical Real Space Reconstruction (IHRSR) program hsearch_lorentz v1.5.

Statistical analysis and plotting were conducted with GraphPad Prism 10.

For manuscripts utilizing custom algorithms or software that are central to the research but not yet described in published literature, software must be made available to editors and reviewers. We strongly encourage code deposition in a community repository (e.g. GitHub). See the Nature Portfolio guidelines for submitting code & software for further information.

Data

Policy information about availability of data

All manuscripts must include a data availability statement. This statement should provide the following information, where applicable:

- Accession codes, unique identifiers, or web links for publicly available datasets
- A description of any restrictions on data availability
- For clinical datasets or third party data, please ensure that the statement adheres to our policy

The cryo-EM density maps and atomic models generated in this study have been deposited in the Electron Microscopy Data Bank (EMDB) and Protein Data Bank (PDB): fascin bound filament 1 (EMDB: EMD-43364; PDB: 8VO5); fascin bound filament 2 (EMDB: EMD-43365; PDB: 8VO6); composite map of the fascin crossbridge (EMDB: EMD-43366; PDB: 8VO7); multibody binned reconstructions eigen_left: (EMDB: EMD-43367; PDB: 8VO8); eigen_middle: (EMDB: EMD-43368; PDB: 8VO9); eigen_right: (EMDB: EMD-43369; PDB: 8VOA); fascin crosslinked hexagonal bundle element with a box size of 460 Å (EMDB: EMD-43370); fascin crosslinked hexagonal bundle element with a box size of 740 Å (EMDB: EMD-43371); subtomogram average of fascin crossbridge (EMDB: EMD-43372). The trained neural networks for denoising and semantically segmenting micrographs and tomograms as well as the atomic models and volume maps used to generate synthetic datasets are available at Zenodo: https://doi.org/10.5281/zenodo.10456803. All other data are presented in the manuscript.

Research involving human participants, their data, or biological material

Policy information about studies with human participants or human data. See also policy information about sex, gender (identity/presentation), and sexual orientation and race, ethnicity and racism.

Reporting on sex and gender	N/A
Reporting on race, ethnicity, or other socially relevant groupings	N/A
Population characteristics	N/A
Recruitment	N/A
Ethics oversight	N/A

Note that full information on the approval of the study protocol must also be provided in the manuscript.

Field-specific reporting

Please select the one below that is the best fit for your research. If you are not sure, read the appropriate sections before making your selection.

☒ Life sciences ☐ Behavioural & social sciences ☐ Ecological, evolutionary & environmental sciences

For a reference copy of the document with all sections, see nature.com/documents/nr-reporting-summary-flat.pdf

Life sciences study design

All studies must disclose on these points even when the disclosure is negative.

Sample size	Sample sizes were not pre-determined. The amount of data collected were limited by the length of cryo-EM imaging sessions. Sufficient data were collected to achieve high-resolution reconstructions which addressed the scientific questions of the study.
Data exclusions	No data were excluded.
Replication	Highly similar reconstructions of the fascin crossbridge were obtained using single particle cryo-EM and subtomogram averaging, using independent methods and datasets.
Randomization	Particles were randomly assigned into half-datasets for resolution assessment by Fourier Shell Correlation analysis.
Blinding	No blinding was performed, as our study did not involve comparisons between different experimental conditions. Sorting into subpopulations was either performed via automated classification techniques, or by rigid-body docking analysis where orientation was determined through maximal crosscorrelation between the fascin crossbridge atomic model and denoised tomograms.

Reporting for specific materials, systems and methods

We require information from authors about some types of materials, experimental systems and methods used in many studies. Here, indicate whether each material, system or method listed is relevant to your study. If you are not sure if a list item applies to your research, read the appropriate section before selecting a response.

Materials & experimental systems

n/a	Involved in the study
☒	☐ Antibodies
☒	☐ Eukaryotic cell lines
☒	☐ Palaeontology and archaeology
☒	☐ Animals and other organisms
☒	☐ Clinical data
☒	☐ Dual use research of concern
☒	☐ Plants

Methods

n/a	Involved in the study
☒	☐ ChIP-seq
☒	☐ Flow cytometry
☒	☐ MRI-based neuroimaging

Plants

Seed stocks	N/A
Novel plant genotypes	N/A
Authentication	N/A
